# Cardiac remodelling and exercise: What happens with ultra-endurance exercise?

**DOI:** 10.1177/2047487320904511

**Published:** 2020-02-13

**Authors:** Cameron Dockerill, Winok Lapidaire, Adam J Lewandowski, Paul Leeson

**Affiliations:** Oxford Cardiovascular Clinical Research Facility, University of Oxford, Oxford, UK

In 490 BC, an Athenian messenger, Pheidippides, was sent by his generals on a 246-km (153-mi) journey to Sparta to seek help fighting against the Persians in the Battle of Marathon. The ancient Greek historian Herodotus wrote that Pheidippides ‘reached Sparta on the very next day after quitting the city of Athens’. Upon reading this, the British Air Force officer and long-distance runner John Foden wanted to find out if this feat was humanly possible. His success in completing the journey on foot within 36 hours led to the establishment of the *Spartathlon*, now considered one of the world’s classic ultra-marathons. Pheidippides is perhaps even more well known for later running approximately 42.2 km, now the regular marathon distance, from Marathon to Athens to announce the Greek victory, before collapsing and dying from exhaustion on the completion of his mission. In this issue of the journal, Christou et al.^1^ report on the acute changes in cardiac structure and function seen in 27 runners who successfully completed the *Spartathlon* race in September 2017 to uncover the cardiac changes that Pheidippides may have experienced following his journey across ancient Greece.

## The effects of exercise on health

Regular exercise is one of the fundamental aspects of a healthy and active lifestyle, promoting optimal cardiovascular and general health. Physicians across a broad spectrum of medical disciplines are prescribing regular exercise training for their patients to treat a number of chronic diseases. Recent UK health guidelines recommend that adults should aim to be physically active every day, accumulating 150 minutes of moderate exercise, such as walking or cycling, or 75 minutes of vigorous intensity exercise (e.g. running) each week. A weight of evidence has shown that the physiological changes associated with regular exercise can be highly effective for the prevention and treatment of coronary artery disease, hypertension, heart failure, obesity, diabetes mellitus and depression,^2,3^ with ongoing studies investigating the additional physiological benefits of these training regimes.^4^ Regular exercise subjects the heart to bouts of haemodynamic stresses such as pressure and volume overload. In order to meet the systemic demand for an increased blood supply at the site of the working muscles during prolonged and repeated exercise, the heart undergoes physiological adaptation. Commonly observed features of highly trained individuals include enlarged left ventricular (LV) and right ventricular (RV) volumes, significantly increased LV wall thickness and cardiac mass and increased left and right atrial size. This cardiac remodelling increases cardiac output and efficiency of contraction, contributing to the beneficial effects of regular exercise on health.

## When can exercise be bad for the heart?

Despite the marked dose-dependent health benefits of moderate exercise, studies have shown that increasing physical activity beyond 60 minutes a day does not have any additional health benefit.^5^ A 15-year observational study of 52,000 adults demonstrated a U-shaped mortality curve, with higher mileage, faster pace and more frequent running not being associated with better survival rates.^6^ Studies have shown that underlying problems in cardiac function within the population can become more evident during exercise, even in apparently healthy individuals,^7^ and excessive endurance exercise can lead to adverse structural cardiac remodelling. An ultra-marathon is classed as any race that is longer than the 42.2-km distance of a regular marathon race distance. The study population for this report by Christou et al.^1^ had a mean finishing time for the *Spartathlon* of 33 hours, 34 minutes and 27 seconds (2014 minutes). Therefore, the *Spartathlon* race alone represents 27 times the weekly vigorous exercise recommendations, demonstrating the enormity of this ultra-marathon. This study adds significant value, therefore, to our understanding of the impact of a single episode of extreme vigorous exercise over a relatively short timeframe with few, if any, previous studies. The findings of the study provide a fascinating insight into the acute cardiac effects of competing in a *Spartathlon.* Furthermore, it provides some insight as to which pre-event characteristics are related to this acute remodelling during the 246-km running race. The most striking find is an increase in LV wall thickness after this 34-hour period. Clearly, this does not represent typical cardiac remodelling as there has not been sufficient time for myocardial hypertrophy. Notably, the change in wall thickness was less pronounced in those athletes with increased pre-event wall thickness, higher training volumes and less severe dehydration. The authors postulate this acute change in wall thickness, therefore, could relate to factors such as oedema or acute inflammation. Results also showed dilatation and altered systolic function of the RV after the race, but additional blood biomarker analysis in a small subgroup showed no correlation between these cardiac changes and troponin I levels. This suggests that changes may not be directly associated with myocardial necrosis, though larger studies with additional blood biomarker analysis are required to confirm this.

## What can we learn from the *Spartathlon* study?

The study population had a typical training regime of running 110 km per week over a median training period of 10 years. Qualification requirements for the *Spartathlon* include completion of a previous race of at least 100 km and one of at least 200 km within certain time limits. The study population is, therefore, a highly selected group who have previously demonstrated that they can perform repeated ultra-endurance exercise and the acute cardiac impact of ultra-endurance exercise in non-ultra-endurance athletes remains unknown. It is possible the ability of the hearts of these athletes to respond in this way is actually an adaptive response that enables their exercise capacity and limits any adverse effects on the heart. However, this hypothesis is not supported by the finding that less severe acute cardiac remodelling was observed in the participants with a higher pre-marathon training load. Although the study reports the acute cardiac effects of this ultra-marathon alone, the long-term cardiac remodelling that occurs during the competitors’ training for previous and future ultra-marathon events remains unclear. The true underlying reason for the acute changes also remains unclear. Repeated imaging, whether by echocardiography or cardiac magnetic resonance (CMR) imaging, was not performed in the ultra-athletes studied. This additional step could have added further information about the reversibility of these acute changes and whether some of the cardiac remodelling observed in this study had longer-term cardiac implications. CMR imaging would have allowed more precise myocardial characterisation, including assessment of the contribution of myocardial oedema, as previously reported in post-marathon studies,^8^ or alterations in myocardial perfusion. CMR imaging could also have allowed more sophisticated computational analysis of cardiac geometric variation after ultra-marathons to understand whether this differs from typical patterns of remodelling,^9,10^ and given insight into the extent that myocardial fibrosis limits the cardiac changes, or starts to develop after ultra-endurance exercise.

## Future directions

In summary, the study reports acute remodelling and reduced cardiac function in ultra-endurance athletes following the *Spartathlon* ultra-marathon. This does not appear to relate to myocardial necrosis but may well be attributed to oedema or an inflammatory response that would be expected to be reversible. Therefore, while Pheidippides may have experienced some acute cardiac remodelling as a result of his journey, it is unlikely that his death was due to cardiac injury. That said, unlike Pheidippides, *Spartathlon* competitors do not have to make the additional return journey home to deliver news of their victory!.
